# Editorial: Host-cell pathways modulated by influenza virus infection: new insight into pathogenetic mechanisms and cell-targeted antiviral strategies

**DOI:** 10.3389/fcimb.2024.1372896

**Published:** 2024-02-16

**Authors:** Marta De Angelis, Paola Checconi, David Olagnier

**Affiliations:** ^1^Laboratory of Virology, Department of Molecular Medicine, Sapienza University, Rome, Italy; ^2^Laboratory Affiliated to Istituto Pasteur Italia-Fondazione Cenci Bolognetti, Department of Public Health and Infectious Diseases, Sapienza University, Rome, Italy; ^3^Department of Human Sciences and Quality of Life Promotion, San Raffaele University, Rome, Italy; ^4^Laboratory of Microbiology, Scientific Institute for Hospitalization and Health Care, IRCCS San Raffaele Roma, Rome, Italy; ^5^Department of Biomedicine, Aarhus University, Aarhus, Denmark

**Keywords:** influenza virus, host-virus interactions, oxidative stress, inflammation, host-cell targeted therapy

Influenza virus remains a formidable global health threat, causing seasonal epidemics and sporadic pandemics with significant morbidity and mortality where genetically different strains keep emerging through genetic reassortment (Uyeki et al., 2022). Influenza A Virus (IAV) interferes with various host factors and pathways to ensure its replication in cell (Peacock et al., 2019). Conversely, restrictive host factors have the ability to control or limit infection (Long et al., 2019). Understanding the intricate interplay between the virus and host cell is fundamental to unraveling the underlying pathogenetic mechanisms and developing targeted antiviral strategies. This study embarks on a comprehensive exploration of host-cell responses to IAV, seeking novel insights into the cellular and molecular events governing viral replication, host immune evasion, and the consequent development of influenza-associated pathogenesis.

Among the tactics employed by IAV to influence host cell machinery in its favor, it is established that the adjustment of redox-related factors serves as a primary mechanism supporting its replication within the host cell (Khomich et al., 2018). Recent evidence highlights the upregulation of glutaredoxin 1 (GRX1) during influenza infection and inhibiting this factor has been demonstrated to reduce viral replication (Checconi et al., 2023). Conversely, suppressing the NRF2-mediated antioxidant response has been shown to enhance IAV infection (De Angelis et al., 2022). In line, the Proteasome Subunit Alpha type 2 (PSMA2), a highly expressed cellular protein in IAV-infected cells, is found to be essential for IAV to escape viral clearance by facilitating NRF2-mediated reactive oxygen species (ROS) neutralization (Rashid et al., 2022). Notably, the NRF2 transcription factor also governs cytosolic nucleic acid sensing and innate immune responses to viruses (Olagnier et al., 2018).

This Research Topic summarizes some recent advancements in the field, consolidating emerging knowledge regarding host-cell pathways modulated during IAV infection. By elucidating the intricate cellular responses, we aim to shed light on the nuanced aspects of influenza pathogenesis, thereby paving the way for the development of more effective and targeted antiviral strategies. The characterization of molecular mechanisms involved in the modulation of redox state in viral infection and host response is of great relevance to understanding the pathogenetic events underlying such infection and associated complications such as excessive inflammatory response. In addition, since influenza viruses are constantly changing, a problem associated with the use of drugs targeting viral particle/proteins is the possible generation of resistance to antivirals. The identification of intracellular factors affected by influenza virus may identify alternative antiviral strategies defined as “host-cell targeted” approaches able to interfere with viral replication. In this current Topic, various aspects of the host response to influenza virus infection were explored. The emphasis lies in identifying redox-regulated host factors and molecules that undergo modulation upon IAV infection. The goal is to understand their impact on controlling the virus life cycle, shaping the cellular response to infection, and pinpointing potential targets for the development of antiviral and anti-inflammatory drugs.

In the first article, Guo et al. studied the potential role of Resolvin D1 (RvD1), a lipid mediator derived from omega-3 polyunsaturated fatty acid, in protecting the airway barrier during influenza virus infection. The study demonstrated that RvD1 protects against oxidative damage induced by H3N2 virus in airway epithelial cells. Moreover, RvD1 alleviates lung damage in IAV-infected mice. The mechanisms underlying this event is mediated by the activation of NRF2 pathway that in turn inhibits ROS production. This study does not only demonstrate the contribution of oxidative stress and the alterations in antioxidant response in IAV pathogenesis, but also identifies RvD1 as a possible anti-influenza therapeutic option.

In the manuscript published by Ji et al., the interactions between NS1 viral protein and host innate immunity are reviewed, focusing on the interferon pathway. In addition to describing the structural and biological characteristics of NS1 protein, the authors described different molecular mechanisms through which it can exert its activity. It can target RIG-I, regulate the production of cellular mRNA and in both ways, it inhibits the dsRNA-mediated host antiviral pathway and IFN production. NS1 can also interact with ISGs to antagonize the host’s antiviral response. Understanding the role of NS1 in regulating important host cell pathways to counteract viral infection may pave the way for the identification of new antiviral targets.

Charman et al. identified TRIM22 (TRIpartite Motif 22) to confer a pre-existing intracellular defense against influenza virus infection in the respiratory tract. Indeed, its expression is independent by viral infection or innate immune stimulation and the constitute levels are sufficient to inhibit replication acting as intracellular restriction factor of IAV. These results reveal a tissue and cell-type dependent expression of immune genes that play a key role as cellular restriction factor of IAV infection.

In the latest work published in this Research Topic, the role of NOX4 (NADPH oxidase 4) in endothelial cells was investigated. Indeed, endothelial cells are critical for immune activation in lung. Hendricks et al. explored NOX4 influence on airway/lung inflammation and morbidity during IAV infection. The authors demonstrated that endothelial NOX4 ameliorates symptoms of IAV infection related to oxidative stress and inflammation. NOX4 is protective against influenza morbidity and it is a potential target for limiting IAV virus-induced lung inflammation. Understanding the dynamics of NOX4 activity during IAV infection is important for the development of future therapeutic options targeting oxidative stress pathways.

In conclusion, this Research Topic delves into the complex relationship between influenza virus and host cellular pathways, aiming to unravel the molecular intricacies that govern viral replication, host immune evasion, and influenza-associated pathogenesis. By exploring the modulation of redox-regulated factors and cellular responses during infection, the study provides valuable insights into the development of targeted antiviral strategies. The identified host factors, such as GRX1, PSMA2, NRF2, and TRIM22, play critical roles in either facilitating viral replication or serving as intrinsic defense mechanisms. Notably, the exploration of therapeutic options like RvD1 and NOX4 highlights potential avenues for intervention against IAV infection, with implications extending to other respiratory viruses and emerging pathogens. These findings underscore the importance of understanding host-virus interactions at the molecular level for developing effective antiviral and anti-inflammatory strategies and they may inform future investigations into similar mechanisms implicated in infections caused by diverse respiratory viruses, paving the way for the development of broad-spectrum antiviral interventions. As we navigate the challenges posed by evolving viruses, this knowledge becomes increasingly vital for advancing our capabilities in combating respiratory infections and safeguarding global public health.

## Author contributions

MDA: Writing – original draft, Writing – review & editing. PC: Writing – original draft, Writing – review & editing. DO: Writing – original draft, Writing – review & editing.
